# Successfully Treated Norovirus- and Sapovirus-Associated Diarrhea in Three Renal Transplant Patients

**DOI:** 10.1155/2018/6846873

**Published:** 2018-11-12

**Authors:** Noha Ghusson, Gustavo Vasquez

**Affiliations:** Sidney Kimmel Medical College at Thomas Jefferson University, Division of Infectious Diseases, 1015 Chestnut Street, Suite 1020, Philadelphia, PA 19107, USA

## Abstract

**Objectives:**

To examine the burden of norovirus- and sapovirus-related diarrhea in renal transplant patients and to propose the use of nitazoxanide as a therapeutic option for treatment.

**Methods:**

We reviewed three renal transplant patients with viral diarrhea requiring hospitalization due to acute renal failure and signs of graft rejection. All three patients were treated with nitazoxanide. We examined their clinical courses after therapy and compared time to resolution of symptoms and viral shedding.

**Results:**

In all three renal transplant patients, improvement of diarrheal illness was witnessed within one week of nitazoxanide initiation.

**Conclusions:**

Infectious diarrhea remains an underestimated yet significant cause of morbidity in solid organ transplant patients. Norovirus and sapovirus are often responsible for this presentation. Nitazoxanide was used as a treatment modality with success in reduction of symptoms, decreased duration of illness, and cessation of viral shedding.

## 1. Introduction

Sapovirus and norovirus are frequent causes of self-limited diarrheal illnesses in healthy hosts. In solid organ transplant patients, however, these viral infections are a frequent cause of chronic and intermittent diarrhea [1], causing significant morbidity and often requiring hospitalization in this population. With that comes an increased risk for serious disease and an inherent risk of dehydration, acute renal failure, and lower graft/patient survival [1]. Traditionally, immunosuppressant medications are tapered in this clinical scenario as a means to resolve these viral infections. However, decreasing immunosuppressants increase the risk of acute transplant rejection. If an immunosuppressed patient does overcome the diarrheal illness, they remain at risk of prolonged asymptomatic viral shedding [2], thus putting themselves and others at risk of reinfection. Here we discuss three cases of renal transplant patients with diarrhea and the use of nitazoxanide in the treatment of norovirus or sapovirus gastroenteritis.

## 2. Case Presentation

Our first patient is a 30-year-old female with a medical history of systemic lupus erythematosus (SLE) complicated by stage V lupus nephritis. She underwent deceased donor renal transplant in 2005 and has been on chronic immunosuppression with tacrolimus 2 mg twice daily and mycophenolate mofetil 500 mg twice daily for approximately 10 years prior to presentation. Ten years after transplant, the patient presented with a chief complaint of diarrhea consisting of 4-5 loose, watery bowel movements daily. Her symptoms were accompanied by mild nausea, anorexia, and abdominal cramping, as well as an unintentional 15-pound weight loss. On admission, she did not have leukocytosis (WBC 4.5 B/L), but she was anemic with a hemoglobin count of 6.1 g/dL and had an elevated creatinine level of 1.6 mg/dL from 1.1 mg/dL baseline. An extensive workup for diarrhea was performed which revealed a positive fecal lactoferrin, normal fecal fat content, and antitissue transglutaminase antibody, a negative *Clostridium difficile* stool toxin, and a positive stool infectious panel PCR for sapovirus. The patient was treated initially with intravenous fluids and antidiarrheal medications. Mycophenolate mofetil dose was decreased gradually and eventually discontinued. Azathioprine was started in place of mycophenolate mofetil, and the patient did have some improvement in the consistency of her stools but the overall large volume output persisted. Nitazoxanide 500 mg orally (PO) twice daily was initiated, and the patient's diarrhea improved within 3 days. She was treated for 7 days total with this medication, and a repeat stool infectious panel 1 month after therapy demonstrated a negative sapovirus PCR.

Our second patient is a 70-year-old female with end-stage renal disease (ESRD) secondary to after streptococcal glomerulonephritis who received a kidney transplant in 2011. The patient was maintained on tacrolimus 4 mg twice daily, prednisone 5 mg daily, and mycophenolate mofetil 500 mg daily. She presented four years after transplant with intermittent diarrhea for 4 months duration and significant weight loss. On presentation, her creatinine was 3.4 mg/dL from a baseline of 1.2 mg/dL on prior studies. The patient was found to have a positive stool infectious panel PCR for norovirus during an inpatient hospital evaluation. *Clostridium difficile* study was negative, and fecal fat content was within normal limits. Mycophenolate mofetil was discontinued due to concern that her symptoms may be a result of medication side effect. She was subsequently started on azathioprine 50 mg daily for immunosuppression. With no real improvement in symptoms, she was treated with a 3-day course of 500 mg PO nitazoxanide twice daily with modest improvement. Two months later, the patient's symptoms reoccurred, and she received a second, longer course of nitazoxanide for 3 weeks. Her symptoms resolved within a week of treatment but again relapsed over the subsequent 3 months. Stool biofire PCRs were persistently positive for norovirus when checked monthly over the following 5-month time period. Finally, she was started on a 3-week course of nitazoxanide high dose (500 mg PO every eight hours). Initially, this was difficult to tolerate due to gastrointestinal distress, and the frequency of administration was decreased to twice daily. The patient completed the three weeks of nitazoxanide with resolution of her gastrointestinal symptoms. She has been asymptomatic since the prolonged course, and repeat stool PCR for norovirus 10 days after completion of therapy is negative.

The last patient is a 78-year-old male who underwent renal transplant in 2014 due to ESRD as a result of severe diabetic nephropathy. The transplant was complicated by interstitial fibrosis and tubular atrophy, requiring high doses of immunosuppressant medications. These medications included mycophenolate mofetil 500 mg twice daily, tacrolimus 4 mg twice daily, and prednisone 10 mg daily. The patient presented to our hospital 3 years after renal transplant with an evidence of altered mental status, lethargy, and progressive weight loss. Per his family, he was having significant diarrhea associated with agitation and confusion. On admission, he had severe metabolic acidosis and an elevated creatinine level of 7.8 mg/dL from baseline of 2.7 mg/dL, which required urgent hemodialysis. His metabolic derangements improved, but he remained confused. The patient was having significant watery stool output. Infectious workup was negative for *Clostridium difficile* infection but confirmed norovirus on a stool infectious panel. Initially, the immunosuppressant medications, primarily mycophenolate mofetil, were held in hopes of improving his diarrhea. When symptoms did not dissipate, he was started on oral nitazoxanide 500 mg twice daily empirically. The patient's diarrhea did improve within 72 hours of nitazoxanide initiation, and he was treated with a full 14-day course. Simultaneously, the patient developed acute transplant rejection which was confirmed on renal ultrasound. This was thought to be due to decrease in immunosuppressant medications. Since that time, hemodialysis has been reinitiated. Unfortunately, a repeat stool infectious panel after therapy was not collected as the patient was lost to follow-up from both the infectious disease and transplant nephrology clinics.

## 3. Discussion

Sapovirus and norovirus are small, nonenveloped, single-stranded RNA viruses within the Caliciviridae family [2]. These enteric infections are typically community acquired and easily transmitted [1] via food-borne contact, person to person, or contaminated environmental surfaces [3]. While norovirus is the most common cause of viral gastroenteritis in adults [4], accounting for 90% of viral gastroenteritis worldwide [2], sapovirus also remains a major cause of enteric infections within the solid organ transplant population. Despite the significance of these infections, there are no data on the exact incidence of norovirus and sapovirus infections in the transplant population. RT-PCR is the gold standard for diagnosis and is typically made through use of a stool infectious panel [5]. Patients often present with abdominal cramping and watery diarrhea that is nonbloody and can be associated with vomiting [3]. Fever is uncommon, and symptoms are usually self-limited in the healthy adult patient. In transplant patients, however, symptoms can last for several months [5], with a mean duration of 8.7 months [1]. As a result, patients are at increased risk for severe dehydration and acute kidney injury.

81% of patients hospitalized with these infections experience acute renal failure [1] as a result of severe dehydration due to diarrhea. Other causes besides dehydration include oxalic nephropathy and villous atrophy [1] as a direct result of gastrointestinal viral invasion. Such side effects greatly increase rates of hospitalization and ultimately graft injury in this group of patients [5].

Another important issue in management of these infections includes the reduction of immunosuppressant medications once a diagnosis of norovirus or sapovirus has been made. Medications such as mycophenolate mofetil and tacrolimus inherently increase the susceptibility to these infections [1]. As such, they are often tapered in attempt to “promote immune system reconstitution to treat the infection” [6], which ultimately puts patients at significant risk for graft rejection and failure. While it is difficult to maintain a balance between managing infection and preventing rejection, [6] our hope is to eliminate this dichotomy by providing an alternative management plan with use of oral nitazoxanide.

Viral gastroenteritis is often managed by replacement of fluids and correction of electrolyte disturbances [4]. Other methods of managing these infections include decreasing immunosuppressant medications which, as stated above, increases risk of graft rejection. Ribavirin has been offered as a potential therapy for norovirus and sapovirus but has shown no significant promise in reduction of symptoms or viral shedding. As a result, it is important to investigate therapeutic alternatives when reduction of immunosuppressants is not a feasible choice. There is anecdotal evidence supporting the benefit of nitazoxanide for management of norovirus and sapovirus in immunocompromised patients.

Nitazoxanide is a thiazolide anti-infective agent [7] that works against anaerobic bacteria, protozoa, and viruses [2]. The proposed mechanism of antiviral activity is the direct targeting of “cellular pathways involved in the synthesis of viral proteins,” thus inhibiting viral replication [4]. The “drug modulates the host antiviral pathway by potentiating the protein kinase activated by double stranded RNA (PKR), an interferon induced effector of cellular antiviral immunity. Activated PKR will halt viral protein synthesis” [8]. As a result, the medication is taken orally and passed through the gastrointestinal tract, addressing viral replication within the intestinal mucosa [4]. Nitazoxanide is noted to have a benign side effect profile, sitting abdominal pain, headache, diarrhea, and nausea [4] as the most common symptoms. It is a pregnancy category B drug [4] and overall is very well tolerated. The most commonly cited dose of nitazoxanide used is 500 mg tablet twice a day. The median time from first dose to resolution of symptoms is 1.5 days [4] in the literature. Amongst our renal transplant patients, all had significantly reduced duration of illness as well as consistency and frequency of bowel movements within 3 days of drug initiation. All had complete resolution of diarrhea by the end of their therapeutic course. Aside from prolonged diarrheal illness, these viruses can continue to be shed within the stool of infected patients long after disappearance of clinical symptoms [9]. Repeat testing in 2 of our 3 patients shows that nitazoxanide also terminated the asymptomatic shedding. Unfortunately, our third patient was lost to follow-up, but obtaining this information can further support use of this proposed therapy. As such, nitazoxanide is a promising safe, therapeutic option for treatment of viral gastroenteritis in renal transplant patients.

## 4. Conclusion

Infectious diarrhea remains an underestimated yet significant cause of morbidity in solid organ transplant patients. Viral etiologies, such as norovirus and sapovirus make up a high percentage of severe, chronic diarrhea and dehydration requiring hospitalization in this population. Preemptive decrease in immunosuppressant medications further puts patients at risk of graft rejection and acute renal failure.

All three renal transplant patients experienced severe, persistent diarrhea associated with abdominal discomfort, bloating, and dehydration. Each patient was found to have a positive stool biofire PCR for either norovirus or sapovirus in the years following their transplant surgery and suffered acute renal failure as a result. Nitazoxanide was used as a treatment modality with success in reduction of symptoms, decreased duration of illness, and cessation of viral shedding.

## 5. Limitations

Despite the successful outcomes demonstrated amongst our patients with the use of nitazoxanide, our study has limitations that will benefit from further investigation. First, the exact prevalence of norovirus and sapovirus is unknown due to lack of appropriate testing. Diarrhea in renal transplant patients is a common result of medication side effect. Mycophenolate mofetil is a known culprit, for example, and therefore, a PCR stool infectious panel may not be sent in time for diagnosis. Thus, the prevalence of viral diarrhea in this population is grossly underestimated and underdetected.

The outcomes of our 3 patients support the benefit of nitazoxanide for therapy of norovirus- and sapovirus-associated diarrhea. The time to resolution of symptoms and clearance of virus remains unknown. Although improvement of diarrhea can be easily reported, immunocompromised patients with viral gastroenteritis can continue to shed the virus in stool for several weeks after resolution of symptoms. This poses the question as to whether we have fully eradicated the infection or if an asymptomatic balance state (or colonization) between host and virus is possible. By defining clearance of infection, we will be able to determine a clear duration of nitazoxanide therapy in future cases of norovirus or sapovirus diarrhea.

A third question we pose is whether there is an additional benefit to decreasing mycophenolate mofetil dose while concurrently treating these infections with nitazoxanide. All three of our patients had some modification in their immunosuppression regimen prior to initiation of nitazoxanide. Although this may simply be a confounding variable, there is room for future research to determine the role of nitazoxanide with or without deescalating immunosuppressants during acute gastroenteritis in transplant patients.

Lastly, there is opportunity for future efforts in vaccine development for viral diarrhea caused by norovirus and sapovirus. With a vaccine, solid organ transplant patients will be placed at even lower risk of all the complications associated with these enteric illnesses. A preventive approach to this common occurrence could be beneficial. In the meantime, nitazoxanide proves to be a promising therapy for norovirus and sapovirus infectious diarrhea in renal transplant, and potentially all, transplant patients.
